# PAM multiplicity marks genomic target sites as inhibitory to CRISPR-Cas9 editing

**DOI:** 10.1038/ncomms10124

**Published:** 2015-12-08

**Authors:** Abba Malina, Christopher J. F. Cameron, Francis Robert, Mathieu Blanchette, Josée Dostie, Jerry Pelletier

**Affiliations:** 1 Department of Biochemistry, McGill University, Montreal, Quebec H3G 1Y6, Canada; 2 McGill Centre for Bioinformatics and School of Computer Science, McGill University, Montreal, Quebec H3G 0B1, Canada; 3 The Rosalind and Morris Goodman Cancer Research Centre, McGill University, Montreal, Quebec H3G 1Y6, Canada; 4 Department of Oncology, McGill University, Montreal, Quebec H3G 1Y6, Canada

## Abstract

In CRISPR-Cas9 genome editing, the underlying principles for selecting guide RNA (gRNA) sequences that would ensure for efficient target site modification remain poorly understood. Here we show that target sites harbouring multiple protospacer adjacent motifs (PAMs) are refractory to Cas9-mediated repair *in situ*. Thus we refine which substrates should be avoided in gRNA design, implicating PAM density as a novel sequence-specific feature that inhibits *in vivo* Cas9-driven DNA modification.

## Abstract (web summary)

A key step for the use of CRISPR-Cas9 in any study is the design of the guide RNA, however the underlying principles are still poorly understood. Here the authors show that multiple protospacer adjacent motif sequences are refractory to efficient targeting and repair.

## Graphical abstract

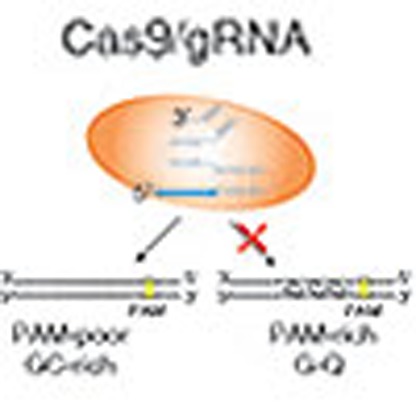

Since its introduction, CRISPR-Cas9 technology revolutionized the field of genome engineering. Derived from a bacterial DNA interference-based acquired immune system, it consists of two essential components, Cas9, an RNA-guided DNA double-strand endonuclease (RGEN), and a small guide RNA (gRNA), that binds to, directs and programs Cas9 to cleave a 20 nucleotide stretch of DNA through base-complementarity, so long as the targeted region is preceded by a short protospacer adjacent motif (PAM) sequence. This inherent simplicity and flexibility in sequence requirements for CRISPR-Cas9 has made genome editing both practical and affordable, and has led to it being adapted for use in a multitude of organisms and for a variety of applications^1^,^2^.

Yet despite its ease of use, predicting *a priori* which gRNAs enable for specific and efficient DNA site targeting by Cas9 has been challenging. Generally, off-target site cleavage can be mitigated by identifying gRNAs that have minimal sequence overlap with other genomic regions or through the co-expression of Cas9 nickase variants that limit the induction of mutagenic repair pathways^3^. However, unlike target site specificity there exist no straightforward guidelines for selecting gRNAs that ensure efficient on target site cleavage. Several large-scale CRISPR-Cas9 library-based screens have sought to evaluate what constitutes an efficient guide sequence, but their results, while valuable and insightful, have either been limited to broad trends in groups of sequences (categorized by GC-content, exonic position, chromatin accessibility and so on)^4^,^5^,^6^,^7^ or have relied on machine-training algorithms whose scoring matrix is unintuitive^8^.

Here, we report that the presence of multiple PAMs within the gRNA target site interferes with efficient Cas9 cleavage and propose that these sites should be avoided to ensure productive DNA editing.

## Results

### PAM-rich TLR reporter sites are refractory to Cas9 editing

**Figure 1:**
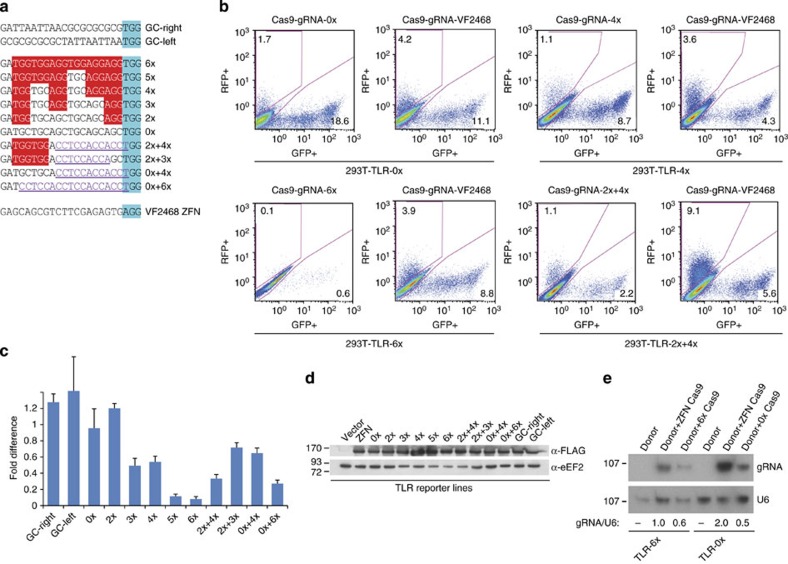
CRISPR-Cas9 genome editing is inhibited by increasing target site PAM density. (**a**) Schematic representation of engineered target sites tested for repair efficiency by CRISPR-Cas9. Blue highlight denotes the target site PAM, red highlights additional PAMs in the target site gRNA homology region, while underlined purple text denotes PAMs on the opposite (and non-homologous to the gRNA) strand. (**b**) TLR flow cytometry analysis. Shown are representative fluorescence scatter plots, with RFP+ cells (NHEJ repair events) versus GFP+ cells (HDR repair events) for the indicated cell lines. One plot is for cells transfected with Cas9 and a gRNA that targets the engineered site compared to a plot for the same cells transfected with Cas9 and gRNA that targets a upstream control site (VF2468). (**c**) Cumulative inhibitory fold-difference in Cas9-driven genome editing efficiency observed at the engineered target site for cell lines expressing TLR loci with increasing number of PAMs relative to the VF2468 control upstream site. *N*=4±s.e.m. (**d**) Cas9 protein levels cannot account for inhibitory fold-difference effects seen in the TLR assay. Expression of Cas9 for the various transfected ‘all-in-one' vectors. Shown is a representative western blot measuring FLAG-epitope expression of the Cas9 protein in the various TLR lines (eEF2 serves as a loading control). (**e**) Differences in gRNA levels cannot account for the inhibitory effects observed in the TLR assay. Northern blot of RNA extracted from the indicated cell lines, each transfected with donor plasmid alone or with Cas9 and the labelled gRNA, either control VF2468 or 0x or 6x gRNAs. The probed transcripts are indicated on the right. Quantification of the fold-difference between the gRNA levels relative to control U6 RNA is shown underneath.

Initially, we were inspired from a recent report by Doudna and co-workers^9^ that delved into the mechanistic details underlying Cas9 enzymatic activity. Two findings struck us as being potential factors in modulating Cas9 target site interactions: (1) the intrinsic affinity of Cas9 to DNA sequences containing multiple PAMs even in the absence of any gRNA complementarity; and (2) the directionality of R-loop formation on the target site initiating at the PAM proximal end of the target region^9^. The latter suggested that thermodynamic asymmetry in the target sequence could promote or impede the rate of R-loop resolution, while the former hinted at a potential bias in sequence discrimination by Cas9 for multiply embedded PAMs in the target region. Conceptually, multiple PAMs could either enhance DNA cleavage by driving and capturing Cas9 to the intended target site or otherwise hinder Cas9's alignment for proper gRNA–DNA interactions. We chose to test these various possibilities *in vivo* by engineering such differences into the previously described traffic light reporter (TLR) system, which is designed to simultaneously measure both non-homologous end joining (NHEJ) repair and homology-directed repair (HDR) pathways^10^,^11^. We engineered TLRs that differed only in their gRNA target sites and would enable us to compare differences between PAM-proximal and PAM-distal GC-rich skewed sequences (labelled ‘GC-right' and ‘GC-left', respectively) as well as target sites containing increasing numbers of PAMs (from zero PAMs (0x) to six PAMs (6x), all of which were controlled for GC-content) (Fig. 1a). A directly upstream VF2468 ZFN target site present in all TLR constructs acted as an internal control. 293T cells, that had stably integrated TLRs at single-copy, were transfected with an ‘all-in-one' vector expressing Cas9 and a gRNA matching the engineered target site^11^, along with a donor GFP repair plasmid. Repair efficiencies were compared with those obtained with a gRNA targeting the VF2468 ZFN target site (Fig. 1b). There was little appreciable difference between GC-skew and target site repair efficiency, with both ‘GC-right' and ‘GC-left' reporters showing only slightly higher repair rates at the engineered site relative to the cognate upstream ZFN target site (Fig. 1c). This is in contrast to reporters with engineered target sites harbouring multiple PAMs: there was a >10-fold difference between 6x and 0x Cas9-modified TLR lines (Fig. 1b,c). (Note that while this effect encompasses the total combined repair efficiencies for NHEJ and HDR repair pathways, their ratios do differ between the upstream ZFN control and engineered GC-rich site. This may reflect differences in sequence requirements between the repair pathways, with GC-rich tracts either promoting or disfavouring one repair pathway versus another, but the exact mechanism for this incongruity is thus far elusive.) This inhibitory effect dissipated as the number of PAMs present in the TLR target site decreased, with the greatest effects being observed for 6x and 5x lines (∼10-fold inhibition), to more moderate effects for 3x and 4x lines (2–2.5-fold inhibition), all relative to the 0x line (Fig. 1b,c). Notably, this effect appeared to require that the PAMs be aligned on the same targeted DNA strand for full inhibition, as constructs where the majority of PAMs were placed on the opposite (and non-complementary) strand of the gRNA, were better substrates for editing by Cas9 (∼threefold inhibition for a target site that had six PAMs, two on the target strand, four on the opposite (2x+4x) or all on the opposite strand (0x+6x); and ∼1.5-fold inhibition for a target site that had five PAMs, two on the target strand and three on the opposite (2x+3x) or four PAMs all on the opposite strand (0x+4x)), suggesting that perhaps what prevents Cas9 editing is more a structural, as opposed to local, DNA sequence arrangement effect (Fig. 1a–c, see also below). Moreover, this consistent difference in editing efficiencies between the various PAM containing TLR lines is not explained by discrepancies in Cas9 expression following transfection between the various constructs (Fig. 1d), nor was it because of reduced transcription from the U6 promoter from potentially interfering secondary structures that could arise from the GC-rich gRNA sequences (Fig. 1e). These results contrast with a recent report where a substantial and consistent enhancement in the rates of editing for substrates seen were for target sites bearing a single NGG directly adjacent to the actual PAM in *Caenorhabditis elegans*^12^. In our hands, we have observed no such enhancement from such sequences (compare ‘2x' with ‘0x' in Fig. 1a–c), although a far greater number of sequences would have to be assayed to conclusively rule out any such position effects in the mammalian setting.

### PAM-rich sites only partially inhibit Cas9 *in vitro*

To gain insight into the molecular basis for the poor editing efficiency obtained with PAM-rich target sequences, we performed *in vitro* binding and cleavage assays with recombinant Cas9 coupled to crRNAs with either 0 PAMs (0x) or 6 PAMs (6x) (in conjunction with a tracrRNA) and incubated in the presence of radiolabelled DNA duplexes containing either zero PAMs (0x) or six PAMs (6x) (Supplementary Fig. 1). Under our assay conditions, we observed a slight overall preference of Cas9 to cleave targets that have a lower density of PAMs. While Cas9 appeared to cleave either 0x or 6x DNA substrates roughly equivalently (and only when coupled to matching crRNAs), this occurred despite it binding ∼twofold less to the 0x DNA substrate (Supplementary Fig. 1). Nevertheless, this minor intrinsic negative ability of Cas9 to cleave PAM-rich substrates cannot fully explain the strong adverse modification observed for targets sequences bearing multiple PAMs by Cas9 *in vivo*. It is conceivable that sequence specificity could influence DNA repair efficiencies, but the fact that both NHEJ and HDR pathways are equally and negatively impacted by increasing PAM density (Fig. 1b) suggests that such inhibitory effects are likely not due to downstream events post-Cas9-driven double-stranded break (DSB) formation. Thus, in addition to slightly reduced cleavage efficiency by Cas9 at sites containing high numbers of PAM, there must be other sequence-specificities arising *in vivo* promoting such effects as well.

### G-Q motifs at endogenous sites impair Cas9 editing *in situ*

**Figure 2:**
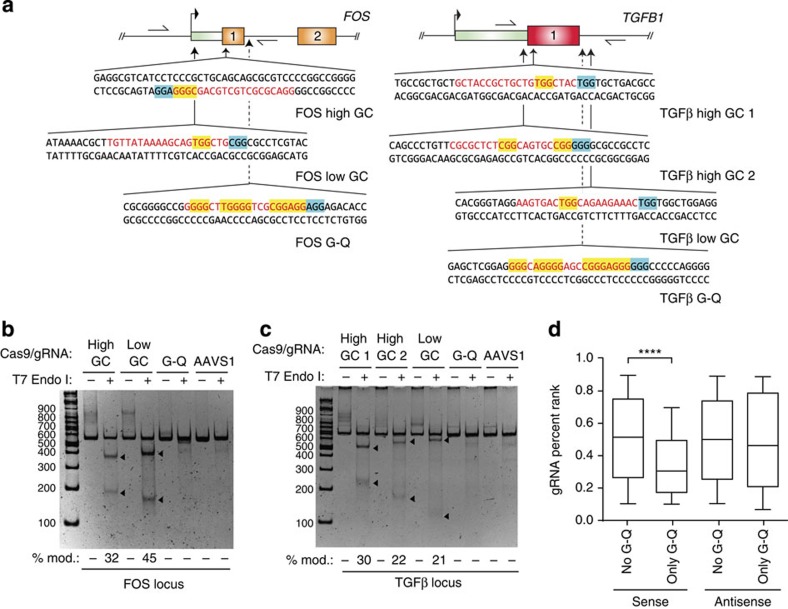
Endogenous PAM-rich/G-Q motif sites are refractory to Cas9-driven editing. (**a**) Schematic representation of the location for each target site sequence in their genic context for *FOS* and *TGFB1* loci. The sequences of the target sites are shown. Red text indicates the gRNA complementary target site sequence, blue highlights the targeted PAM, and yellow highlights NGG motifs within the body of the target site (flanking non-targeted DNA sequences is written in black text). Primer binding sites for PCR amplification prior to performing the T7endI assay are marked as lines with half-arrowheads. (**b**,**c**) Genomic modification by Cas9 at PAM-rich G-Q local sites is strongly inhibited relative to nearby PAM poor low or high GC-content target sites as measured by a T7endI assay. Shown are representative experiments with the digested fragments, denoted by triangles, representing the fraction of the targeted genomic region by Cas9 that had been repaired by NHEJ. The 100 bp increments of the DNA ladder are shown to the left of the blot. Underneath each lane the % modification is indicated. (**d**) Sequences with G-Q motifs on the sense strand are disfavoured by CRISPR-Cas9 for editing. Box-and-whisker plots of the reported gRNA percent ranks separating sequences into those that exclude or include predicted G-Q motifs, for either sense or antisense gRNA targeting site sequences. The top, middle and bottom lines of the ‘box' represent the 25th, 50th and 75th percentiles, respectively, while the ‘whiskers' represent the 10th and 90th percentiles. ****, *P* value ≤0.0001 as determined by the Kolmogorov–Smirnov test.

One intriguing possibility is the propensity for NGG-rich sequence tracts to form higher order DNA tertiary structures such as G-quadruplexes (G-Q)^13^, which might interfere with Cas9's ability to engage in productive endonuclease DNA complexes. To further test this hypothesis in a less artificial setting, we chose to assay Cas9-driven genomic editing on two separate endogenous loci, *FOS* and *TGFB1,* known to contain CpG islands and a relatively high GC-content at early exonic positions^14^. Specifically, we chose sequences that were in the vicinity of one another, and in early regions of the transcribed gene, thus minimizing differences in modification due to variations in chromatin inaccessibility or other epigenetic alterations (Fig. 2a). Sites within each gene were chosen to represent three different kinds of targets: (1) low GC-content containing sequences; (2) high GC-content containing sequences, but with relatively few NGG tracts; and (3) NGG-rich sequences that are predicted to form G-Q structures (Table 1). Following transfection of ‘all-in-one' vectors expressing Cas9 and the corresponding gRNA into 293T cells, target site editing was assessed via the T7 endonuclease I assay (a gRNA targeting the *AAVS1* locus served as a negative control). Strikingly, there was no detectable mutagenesis at any of the G-Q target sites, whereas both ‘high GC' and ‘low GC' gRNAs showed similar mutagenesis efficiencies (Fig. 2b,c). These differences were not attributable to gross variations in transfection efficiencies, as the levels of expression for the various Cas9 constructs were equivalent for all vectors and gRNA combinations (Supplementary Fig. 2). These effects persisted across three distinct cell lines (293T, HeLa and MCF-7), and for multiple different paired loci: G-Q versus high GC regions from PIM1 (Exon 1), TNFA (Exon 4) and, much less reliably, KRAS (Exon 1) (Supplementary Fig. 3 and Table 1), and were not attributable to differences in Cas9/gRNA plasmid expression (Supplementary Fig. 4).

These results are consistent with those from two separate large-scale studies which noted that gRNAs targeting regions of high GC-content (≥80%) collectively ranked poorer as Cas9 substrates^5^,^8^,^15^, a category that should also encompass sequences with multiple PAMs. Thus, we sought to further test whether sub-stratification of gRNA sequences into those predicted to form G-Q structures (which are *de facto*^5^ ‘NGG^3^'-dense) would predict an even greater inhibitory effect by re-analysing the published data set of annotated and ranked gRNAs in Doench *et al*.^8^ (Fig. 2d, Supplementary Fig. 5 and Supplementary Data 1). Isolation of sequences with motifs predicted to form a G-Q demonstrated a significant relative fold-depletion (∼1.7-fold, *P* value <0.0001) when compared to the median-depleted gRNA in this set (Fig. 2d). Moreover, this inhibitory trend was also strand-specific: target sites that had G-Q motifs on the opposite strand showed virtually no difference in repair efficiencies relative to the median target site sequence (Fig. 2d), which is consistent to what was observed in the TLR assay for fold-inhibition by target site PAM multiplicity (Fig. 1c). This effect persisted even when controlling for sequences based on their GC-content, or, simply, by the proportion of G-residues (Supplementary Fig. 3a,b). Notably, when separated into G-Q motif predicted and not predicted pools, most of the previously seen high GC-content negative bias diminished substantially, perhaps reflecting a dependence on PAM density in promoting much of the disparity among reported gRNA sequences (a ∼1.4-fold drop in percent rank, Supplementary Fig. 5a). The fact that only G-rich sequences that can form G-Q motifs were appreciably poorer substrates than those which cannot, implies that it is the configuration itself (of multiple tetrad-forming NGGs) that is driving this inhibitory effect (Supplementary Fig. 5b). Collectively, these results suggest that high GC-content *per se* is not the defining feature that limits Cas9-driven editing, but rather the context of GC-rich sequences—that the density of NGG motifs and their potential to form G-Q structures is what influences the rates of modification. In sum, we have uncovered a novel guideline when selecting DNA sequences for Cas9 gRNAs. By avoiding target sites that contain a high proportion NGG motifs with a potential to form G-Q structures, this simple rule ensures for efficient cleavage even in the context of very GC-rich regions, and one that likely should be incorporated into current design schemes and algorithmic tools.

## Materials and Methods

### Vectors and cell culture, and lentiviral transduction

The pLeGO-Cas9-gRNA (pLC), TLR, Δ20 eGFP donor plasmids were used for Cas9 expression and establishment of the TLR reporter system^11^. 293T/17, HeLa and MCF-7 cells (ATCC) were maintained in DMEM supplemented with 10% fetal bovine serum, 100 U ml^−1^ penicillin/streptomycin and 100 U ml^−1^ of glutamine. Lentiviral transductions were performed using second-generation vectors. Essentially, 10 μg of pLC, 7.5 μg of packaging plasmid psPAX2, and 3 μg of envelope vector (pMDG2) were combined and transfected by calcium phosphate into 293T/17 cells. The medium was changed the following day, and 48 h post transfection, lentiviral supernatant was collected. The lentiviral supernatant (0.5 ml) was mixed with an equal volume of medium and added to a well of a six-well dish containing 293T/17 cells, with 10-fold serial dilutions to ensure a low multiplicity of infection (MOI).

### Traffic light reporter (TLR)

TLR reporter constructs were generated by subcloning custom target sites derived from synthesized oligos (as depicted in Fig. 1a) into the SpeI site of pCVL TL-dsRED Reporter 2.1 (Addgene). 293T cells were then lentivirally transduced with TLR vectors at low MOI, such that following puromycin selection (at 2 μg ml^−1^) only ∼1–5% of the total cell population remained. After expansion, these lines were then transfected using calcium phosphate with the indicated pLC ‘all-in-one' vector and donor Δ20 eGFP that expresses tagBFP fluorescent marker. Cells were passaged over the course of 7 days to allow for genome-editing events to occur, at which point cells were trypsinized and resuspended in phosphate-buffered saline+2% fetal bovine serum before analysing the samples on a Gallios Flow Cytometer (Beckman Coulter). eGFP and dsRED fluorescence was measured using a 488 nm laser for excitation and each detected using a 530/30 filter and 610/20 filter, respectively. mTagBFP fluorescence from the donor plasmid fluorescence was measured using a 405-nm laser for excitation and a 450/50 filter for detection. A total of 100,000 events were collected and analysed using FlowJo software. The fold-difference of the total repair events between the transfections of pLC-gRNA constructs and the control pLC-ZFN constructs in the corresponding TLR lines were estimated by adding the %GFP+ and %RFP+ events in the indicated fluorescent gates in Fig. 1b and normalizing for differences in transfection efficiencies by using BFP+ events. More formally, we used the following formula:


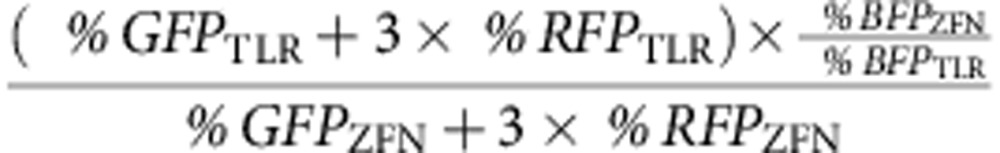


### Western blots

Whole cell extracts were prepared by direct lysis of cells in NuPAGE LDS sample buffer. Total protein concentrations were determined by UV absorbance at 280 nm using a Nanodrop (Thermo Scientific) and equal amounts of total protein (30 μg) was resolved on a 10% Bis-Tris NuPAGE gel in 1 × MOPS buffer and subsequently transferred onto PVDF membrane. Following transfer, membranes were probed with the indicated antibodies, and visualized using enhanced chemiluminescence (Perkin Elmer). The antibodies used were anti-FLAG epitope (M2, Sigma, 1:5,000) and anti-eEF2 (#2332, Cell Signaling, 1:1,000). Full uncropped images are provided in Supplementary Fig. 6.

### Small RNA northern blot

Total RNA (15 μg) was isolated using Trizol (Life Technologies) and resolved on a denaturing 8 M UREA/15% polyacrylamide (19:1, acrylamide:bisacrylamide) 1 × TBE gel. The gel samples were transferred to a Hybond N+ membrane (GE Healthcare) using a semi-dry apparatus (Bio-Rad) at room temperature at 300 mA for 1.5 h in 1 × TBE transfer buffer. Following transfer, the RNA was crosslinked to the membrane using a Stratalinker UV Crosslinker (Stratagene). The membrane was pre-hybridized in buffer containing 0.2 M Na_2_PO_4_ buffer (pH 7)/ 7% SDS for 30 min at 37 °C, at which point 40 × 10^6^ c.p.m. of radioactive probe was added and incubated overnight at 37 °C. Probes were labelled using T4 PNK (NEB) and γ-^32^P-ATP (Perkin Elmer) and unincorporated γ-^32^P-ATP removed by G-25 Sephadex spin columns. The following probes were used: U6 (loading control)—5′-GCAGGGGCCATGCTAATCTTCTCTGTATCG-3′ and gRNA (targeting the scaffold portion of the gRNA) 5′-TTCAAGTTGATAACGGACTAGCCT-3′. After incubation, the membrane was washed twice with 2 × SSPE/0.1% SDS for 30 min. at 42 °C and twice with 0.5 × SSPE/ 0.1% SDS for 30 min at 42 °C. The membrane was exposed to a Phosphorimager screen for a few hours and processed using a Typhoon scanner (GE Healthcare) or exposed overnight at −80 °C to X-ray film. Quantitation was performed using ImageJ (NIH). Full uncropped images are provided in Supplementary Fig. 6.

### Electrophoretic mobility shift and *in vitro* cleavage assays

Recombinant wild-type *Streptococcus pyogenes* Cas9 was expressed from pMJ806 (obtained from Addgene) and purified^16^. TracrRNA was produced by *in vitro* transcription from a PCR-amplified template containing a T7 promoter. All crRNAs were synthesized and high-performance liquid chromatography purified (IDT). DNA target oligonucleotides were synthesized (IDT) and the two strands annealed and gel purified. crRNA sequences were: 6x 5′-GAUGGUGGAGGUGGAGGAGGGUUUUAGAGCUAUGCUGUUUUG-3′ and 0x 5′-GAUGCUGCAGCUGCAGCAGCGUUUUAGAGCUAUGCUGUUUUG-3′. DNA oligos were: 6x_F 5′-tcgactacaagtacatGATGGTGGAGGTGGAGGAGGTGGgcatgaagcgctgacg-3′, 6x_R 5′-cgtcagcgcttcatgcCCACCTCCTCCACCTCCACCATCatgtacttgtagtcga-3′, 0x_F 5′-tcgactacaagtacatGATGCTGCAGCTGCAGCAGCTGGgcatgaagcgctgacg-3′, and 0x_R 5′-cgtcagcgcttcatgcCCAGCTGCTGCAGCTGCAGCATCatgtacttgtagtcga-3′ (upper case denotes target sequence, while lower case denotes flanking non-target sequence). The target duplexes were kinased using T4 PNK (NEB) with γ-^32^P-ATP (Perkin Elmer). Gel shifts and *in vitro* cleavage assays were performed using 1 nM radiolabelled duplexed DNA substrate, 10 nM recombinant Cas9, 20 nM tracrRNA and 20 nM crRNA. Before addition of Cas9 and incubation at 37 °C for 60 min., crRNA, tracrRNA and radiolabelled DNA substrate were first heated to 95 °C and slow-cooled to room temperature. EMSA complexes were resolved on a 5% native 0.5X TBE polyacrylamide gels (19:1; acrylamide/bisacrylamide) containing 5 mM MgCl_2_, while cleavage reactions were resolved on a 10% 8 M UREA denaturing 0.5X TBE polyacrylamide gels (19:1 acrylamide:bisacrylamide) containing 5 mM MgCl_2_^17^. The gels were dried and exposed to a PhosphorImager screen for a few hours and processed using a Typhoon scanner (GE Healthcare) or exposed overnight at −80 °C to X-ray film. Quantitation was performed using ImageJ (NIH). Full uncropped images are provided in Supplementary Fig. 6.

### T7 endonuclease I assay

Genomic DNA was isolated from transfected 293T/17 cells by resuspension of the cell pellet in SNET buffer (50 mM Tris-Cl pH 8.0, 100 mM EDTA, 100 mM NaCl, 1% SDS) with Proteinase K (100 μg ml^−1^) and RNase A (25 μg ml^−1^ ) and incubated for 2 h at 55 °C. The lysate was extracted once with phenol/chloroform (pH 8.0), precipitated in 2 volumes ethanol and the DNA pellet was resuspended in 10 mM Tris-HCl (pH 8.0). Genomic DNA (100 ng) was PCR amplified in a 50 μl reaction volume for 35 cycles, using Hot Start Q5 (NEB) and following the manufacturer's recommended reaction and cycling conditions and using the online calculator to measure primer annealing temperatures. The primers that were used to amplify the genomic region flanking the CRISPR-Cas9-driven mutagenic site were: FOS-F, 5′-AGATTAGGACACGCGCCAAG-3′, FOS-R, 5′-GGGAGCCCCCTACTCATCTA-3′, TGFB1-F, 5′-CTATCTCCTCCTCTCCAAGACCA-3′, TGFB1-R, 5′-ATTTCCGTGGGATACTGAGACAC-3′, KRAS-F, 5′-CCTCCGGGGACCCCTAATTCAT-3′, KRAS-R, 5′-GTGCTCGGAGCTCGATTTTCCT-3′, TNFA-F, 5′-AGCACAGGCCTTAGTGGGATACT-3′, TNFA-R, 5′-GTTCTGGAGGCCCCAGTTTGAAT-3′, PIM1-F, 5′-CTTTACTCCTGGCTGCGGG-3′, PIM1-R, 5′-GGCGAGTCGGAGGACAAC-3′. The single-band PCR product was further purified using Qiaquick spin columns (Qiagen). Genome modification efficiency was then assayed using the T7 endonuclease I enzyme following a published protocol on the manufacturer's website (NEB). Ten microliters of the reaction was resolved on a polyacrylamide 1xTBE gel (29:1 acrylamide:bisacrylamide) and stained with ethidium bromide. Relative band intensities were quantified using ImageJ (National Institutes of Health), using the formula: %modification=100 × (1−(1- fraction cleaved)^1/2^).

### Statistical analysis of the gRNAs depletion data set

All 1,841 gRNAs sequences targeting the CDS of selected genes and their corresponding percent ranks were taken as published in Supplementary Table 7 from Doench *et al*.^8^ For prediction of G-Q tetrad motifs sequences were scored as containing putative G-Q structures if they followed the tetrad pattern of G_x_N_y1_G_x_N_y2_G_x_N_y3_G_x_, where x≥2 and y1, y2, y3 can be any nucleotide of any gap length, with only one gap length among the three being zero in length being permissible^18^. Kolmogorov–Smirnov *t*-test and boxplots were calculated and visualized using Graphpad software package (GraphPad Software, Inc.).

## Additional information

**How to cite this article**: Malina, A. *et al.* PAM multiplicity marks genomic target sites as inhibitory to CRISPR-Cas9 editing. *Nat. Commun.* 6:10124 doi: 10.1038/ncomms10124 (2015).

## Supplementary Material

Supplementary Information Supplementary Figures 1-6

Supplementary Data 1 Data reproduced from Supplemental Table 7 from Doench et al^8^. G-Q motif prediction was determined following the criteria rules in Kikin et al1^8^.

## Figures and Tables

**Table 1:** NGG-rich sequences that are predicted to form G-Q structures.

**Name**	Sequence*	**Chromosome**	**Position**†	**Strand**	**%GC-Content**	**QGRS score**‡	**gRNA score**§
FOS low GC	(T)GTTATAAAAGCAGTGGCTG	14q24	75278793-75278814	negative	40	0	0.2543
FOS high GC	GGACGCGCTGCTGCAGCGGG	14q24	75279023-75279045	positive	80	0	0.0750
FOS G-Q	GGGGCTTGGGGTCGCGGAGG	14q24	75279155-75279177	positive	80	17	0.0870
TGFβ low GC	(A)GAGTGACTGGCAGAAGAAAC	19q13	41352594-41352616	negative	45	0	0.1329
TGFβ high GC 1	GCTACCGCTGCTGTGGCTAC	19q13	41352987-41353009	negative	65	0	0.0443
TGFβ high GC 2	(C)GCGCTCTCGGCAGTGCCGG	19q13	41353059-41353081	negative	80	0	0.0477
TGFβ G-Q	GGGCAGGGGAGCCGGGAGGG	19q13	41352655-41352677	negative	85	39	0.0100
KRAS high GC	(A)GCTGGGAGCGAGCGCGGCGC	12p12	25250809-25250828	positive	85	0	0.1610
KRAS G-Q	(C)GGGCGAAGGTGGCGGCGGCT	12p12	25250860-25250879	positive	80	19	0.0172
TNFα high GC	GGAGACGGCGATGCGGCTGA	6p21	31577299-31577321	negative	70	0	0.0756
TNFα G-Q	(A)GTTGGGGCAGGGGAGGCGTT	6p21	31577560-31577582	negative	70	19	0.0287
PIM1 high GC	(T)GCAGCGCTGCCCGACCCCGC	6p21	37170342-37170361	positive	85	0	0.1501
PIM1 G-Q	GTCGGTGGCAGCGGCGGCGG	6p21	37170220-37170239	positive	85	18	0.0670

gRNA, guide RNA; TNFα, tumour necrosis factor-α.

^*^Brackets denote non-G starting nucleotide found in the genomic sequence which was altered to G in the gRNA sequence for proper U6 transcription initiation.

^†^Position numbers based on the assembly for the human genome GRCh38 from NCBI.

^‡^Based on the scoring system from http://bioinformatics.ramapo.edu/QGRS/ which ranks the likelihood for a given candidate sequence to form a G-Q structure^18^.

^§^Based on the scoring algorithm as reported^8^.
