# Supplementary material for: PAM multiplicity marks genomic target sites as inhibitory to CRISPR-Cas9 editing
Source: Nat Commun. 2015 Dec 8;6:10124. doi: 10.1038/ncomms10124 (PMC4686818; doi:10.1038/ncomms10124)
Supplement: Supplementary Information — Supplementary Figures 1-6 [file ncomms10124-s1.pdf]

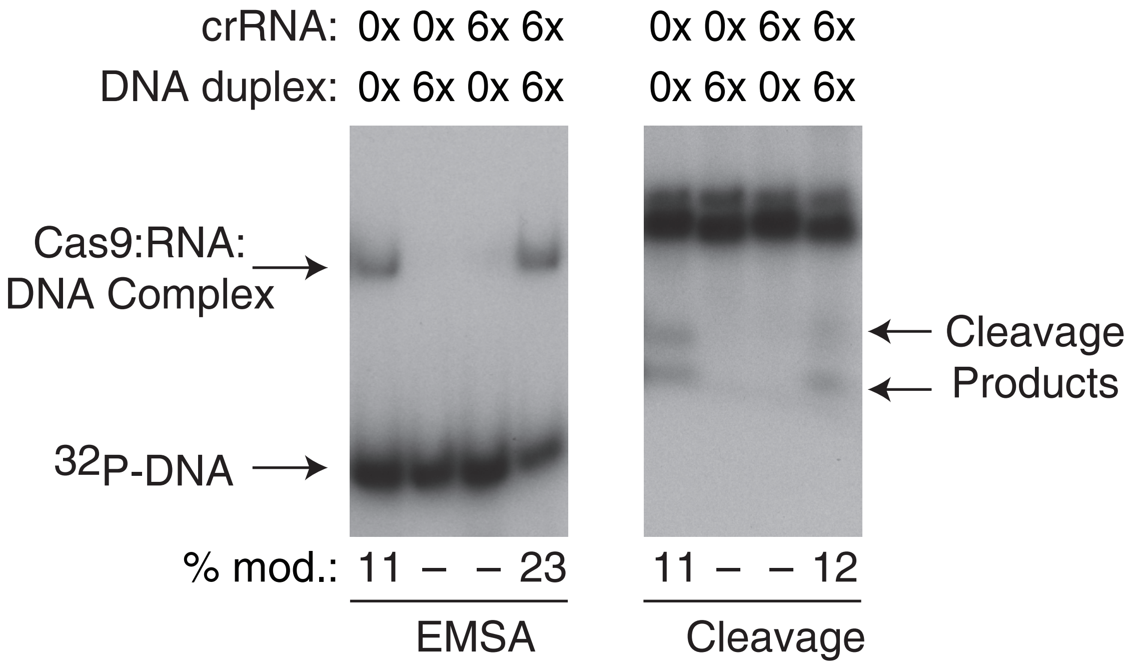


**Supplementary Figure 1. Target sites containing 6xPAMs are only partially inhibitory to intrinsic Cas9 binding and cleavage activities** Shown are representative in vitro EMSA (left) and cleavage assays (right) using recombinant Cas9 programmed with tracrRNA, the indicated crRNA, and radiolabeled DNA duplex substrate. DNA cleavage products are indicated by the arrows. Quantitation of the % binding or % cleavage of programmed Cas9 for the various DNA substrates is shown below.

**SUPPLEMENTARY FIGURE-1_Pelletier**

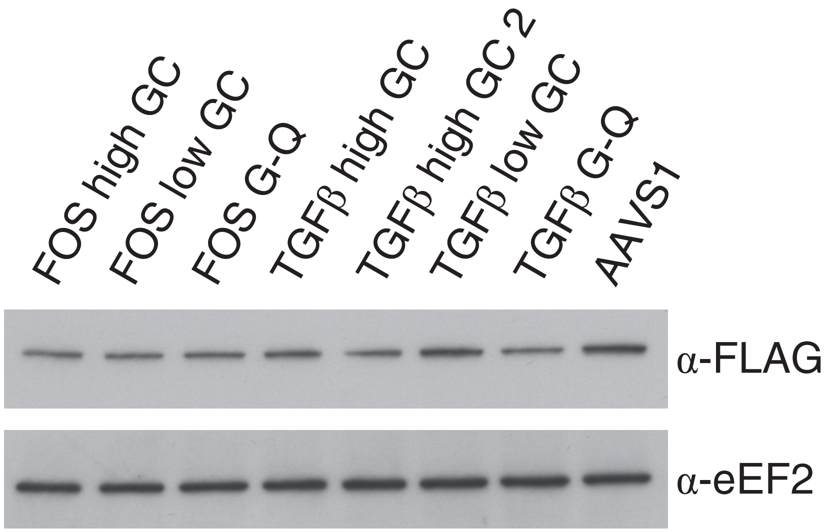


**Supplementary Figure 2. Expression levels of Cas9** Shown is a Western blot measuring FLAG-epitope expression of the Cas9 protein from a representative experiment from Figs. 2b and 2c (eEF2 serves as a loading control).

**SUPPLEMENTARY FIGURE-2_Pelletier**

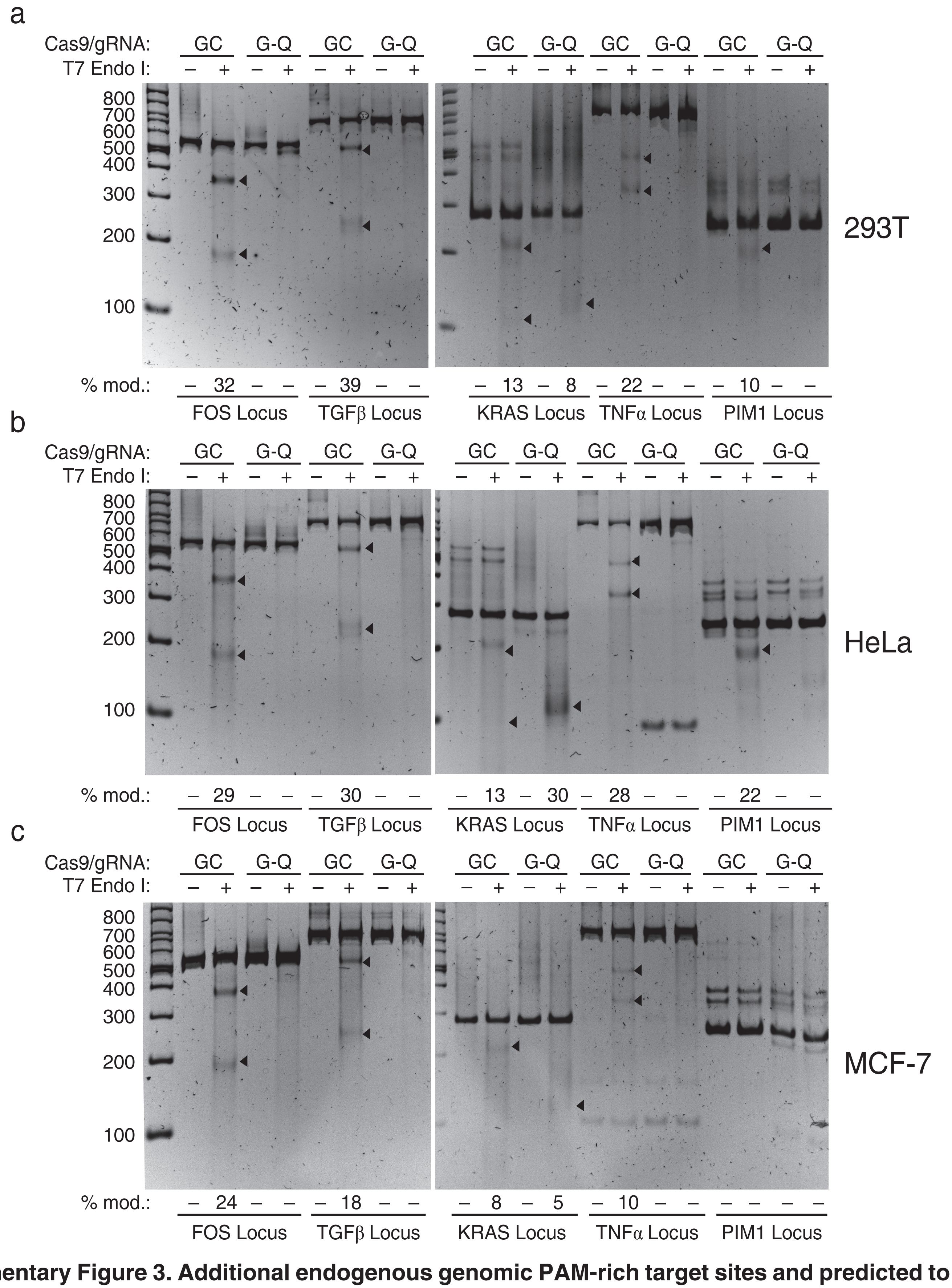


**Supplementary Figure 3. Additional endogenous genomic PAM-rich target sites and predicted to form G-Q structures are refractory to Cas9-driven modification** (**a-c**) Genomic modification by Cas9 at PAM-rich G-Q local sites (denoted by "G-Q") is strongly inhibited relative to nearby PAM poor high-GC content target sites (denoted by "GC") as measured by a T7endI assay. Shown are representative experiments with the digested fragments, denoted by triangles, representing the fraction of the targeted genomic region by Cas9 that had been repaired by NHEJ. The 100 bp increments of the DNA ladder are shown to the left of the blot. Underneath each lane the % modification is indicated. Note that in (**c**), PIM1 modification in MCF-7 is below the limits of detection for the assay, possibly the result of slightly weaker plasmid expression for that line (see Supplementary Figure 4c).

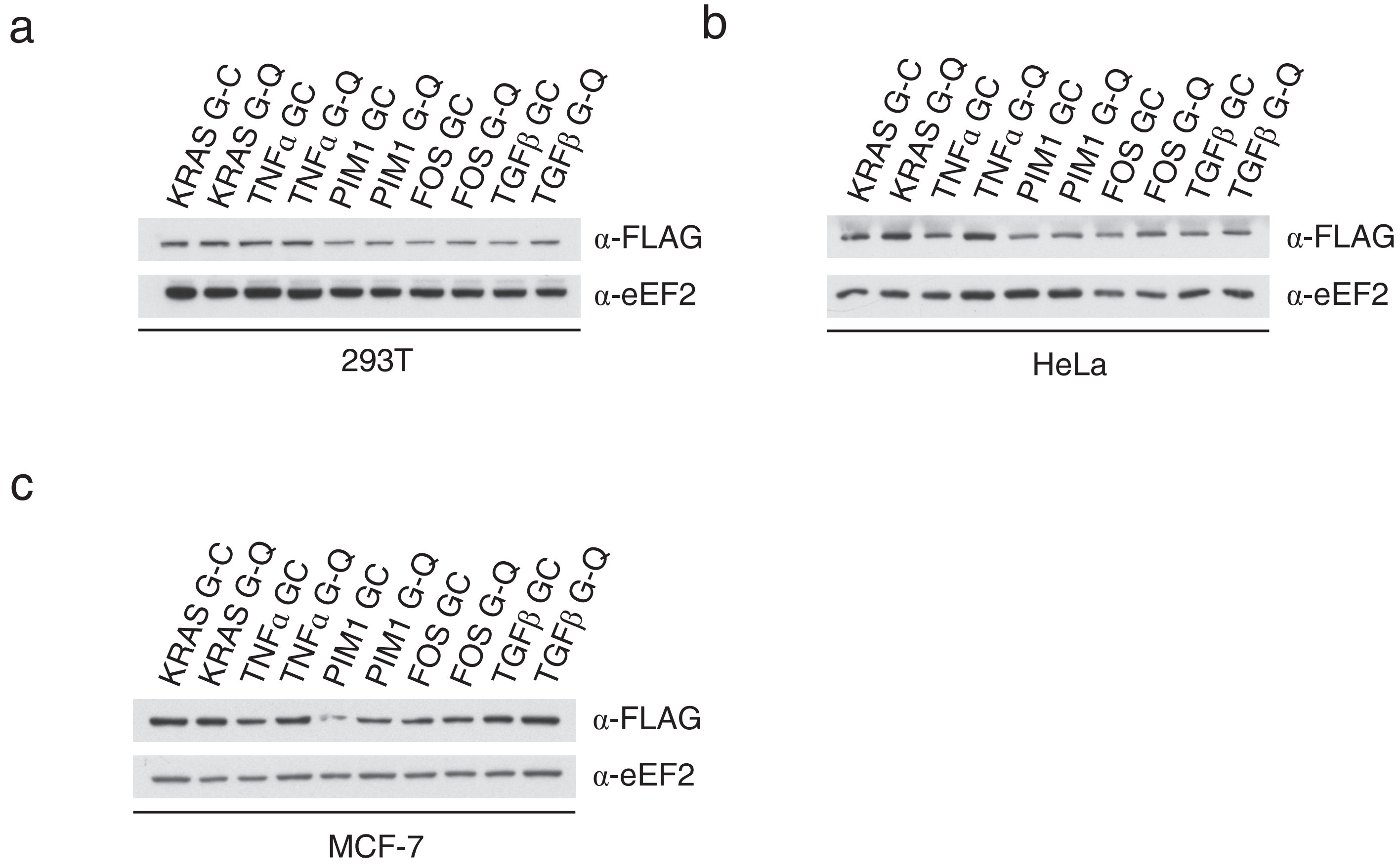


**Supplementary Figure 4. Expression levels of Cas9** (**a-c**) Shown is a Western blot measuring FLAG-epitope expression of the Cas9 protein in each of indicated cell lines from a representative experiment from Supplementary Figure 3 (eEF2 serves as a loading control).

**SUPPLEMENTARY FIGURE-4_Pelletier**

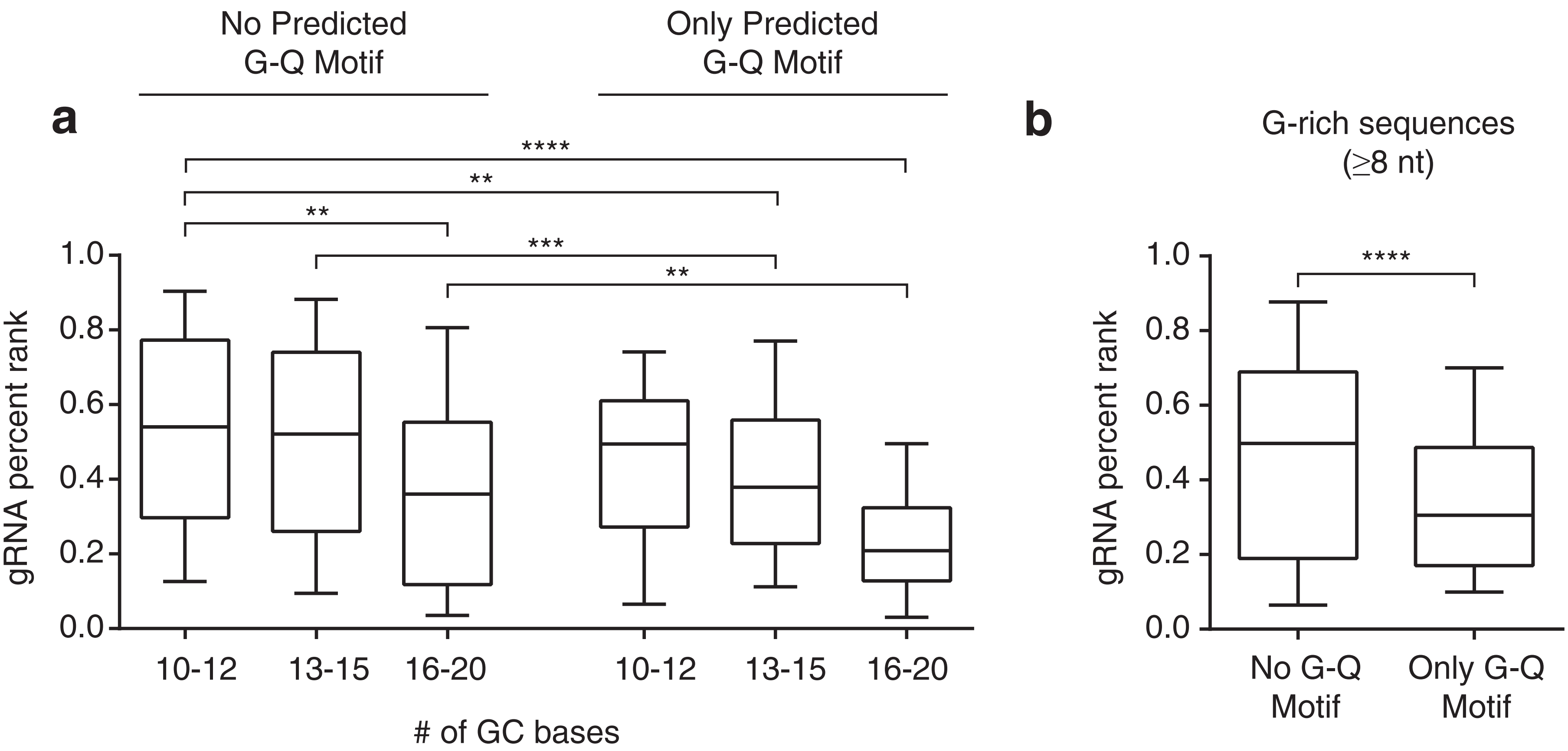


**Supplementary Figure 5. G-Q motifs in gRNA sequences are better predictors of poorer CRISPR/Cas9 substrates than GC-content alone** (**a**) Box-and-whisker plots of the reported gRNA percent ranks separating sequences into those that exclude or include predicted G-Q motifs and sorting them by the number of G and C bases from Doench et al 8. The top, middle and bottom lines of the "box" represent the 25th, 50th and 75th percentiles, respectively, while the "whiskers" represent the 10th and 90th percentiles. ****, P value ≤ 0.0001; **, P value ≤ 0.01; as determined by the Kolmogorov-Smirnov test. (**b**) Box-and-whisker plots of the reported gRNA percent ranks of only the subset of G-rich sequences (≥8 nt), separating the sequences into those that exclude or include predicted G-Q motifs of G and C bases. The top, middle and bottom lines of the "box" represent the 25th, 50th and 75th percentiles, respectively, while the "whiskers" represent the 10th and 90th percentiles. ****, P value ≤ 0.0001; **, P value ≤ 0.01; as determined by the Kolmogorov-Smirnov t-test.

**SUPPLEMENTARY FIGURE-5_Pelletier**

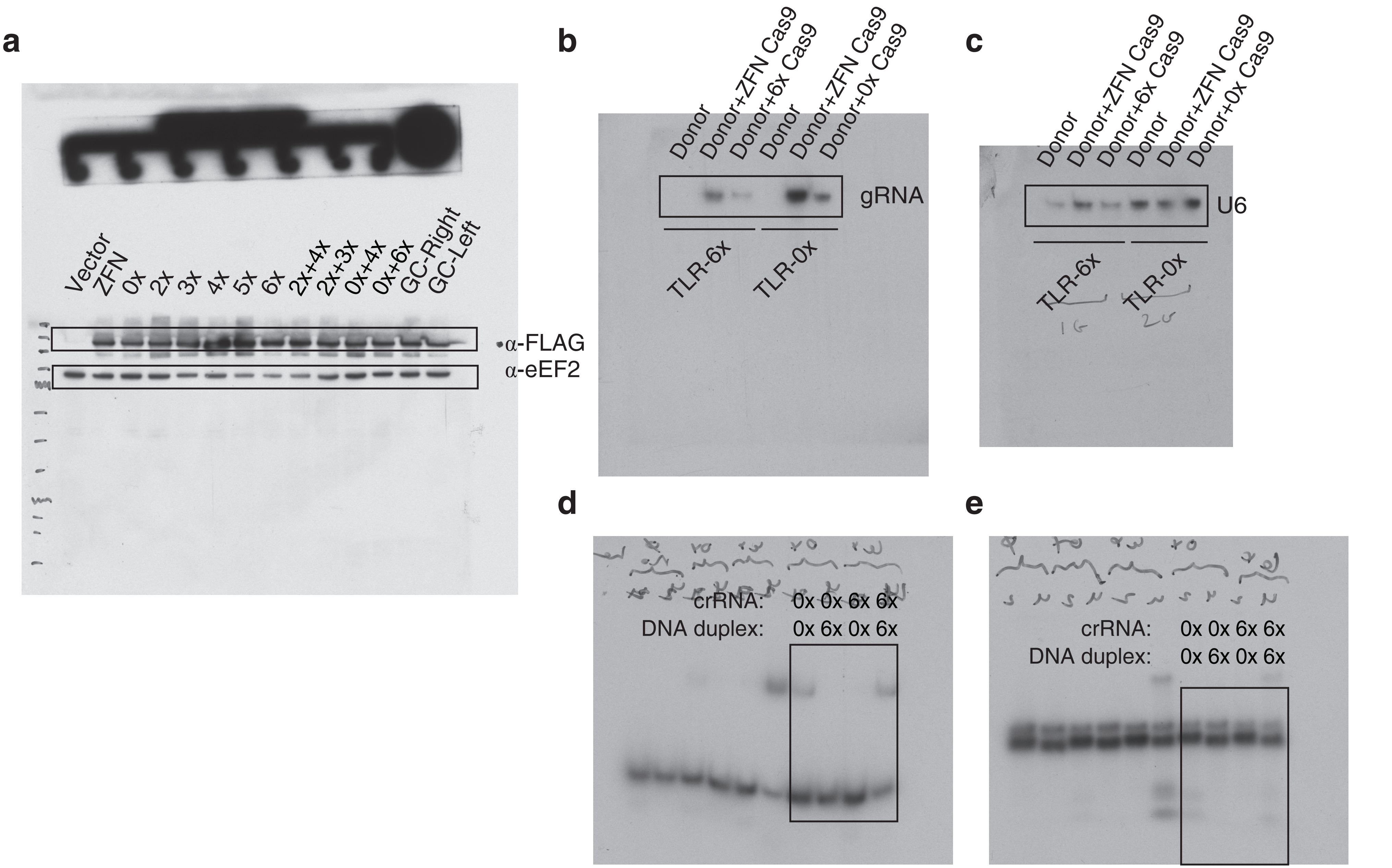


**Supplementary Figure 6. Uncropped scans** (**a**) Corresponds to Figure 1d. (**b-c**) Corresponds to Figure 1e. (**d-e**) Corresponds to Supplementary Figure 1.

**SUPPLEMENTARY FIGURE-6_Pelletier**
